# Correlates of video games playing among adolescents in an Islamic country

**DOI:** 10.1186/1471-2458-10-286

**Published:** 2010-05-27

**Authors:** Hamid Allahverdipour, Mohsen Bazargan, Abdollah Farhadinasab, Babak Moeini

**Affiliations:** 1 Department of Public Health and Management, School of Public Health & Nutrition, Tabriz University of Medical Sciences, Tabriz, Iran; 2 Department of Family Medicine, Charles Drew University of Medicine and Science, Los Angeles, California, USA; 3 Department of Psychiatry, Shahid Beheshti University of Medical Sciences, Tehran, Iran; 4 Research Institute of Behavioral Disorders & Substance Abuse, and Department of Public health, Hamadan University of Medical Sciences, Hamadan, Iran

## Abstract

**Background:**

No study has ever explored the prevalence and correlates of video game playing among children in the Islamic Republic of Iran. This study describes patterns and correlates of excessive video game use in a random sample of middle-school students in Iran. Specifically, we examine the relationship between video game playing and psychological well-being, aggressive behaviors, and adolescents' perceived threat of video-computer game playing.

**Methods:**

This cross-sectional study was performed with a random sample of 444 adolescents recruited from eight middle schools. A self-administered, anonymous questionnaire covered socio-demographics, video gaming behaviors, mental health status, self-reported aggressive behaviors, and perceived side effects of video game playing.

**Results:**

Overall, participants spent an average of 6.3 hours per week playing video games. Moreover, 47% of participants reported that they had played one or more intensely violent games. Non-gamers reported suffering poorer mental health compared to excessive gamers. Both non-gamers and excessive gamers overall reported suffering poorer mental health compared to low or moderate players. Participants who initiated gaming at younger ages were more likely to score poorer in mental health measures. Participants' self-reported aggressive behaviors were associated with length of gaming. Boys, but not girls, who reported playing video games excessively showed more aggressive behaviors. A multiple binary logistic regression shows that when controlling for other variables, older students, those who perceived less serious side effects of video gaming, and those who have personal computers, were more likely to report that they had played video games excessively.

**Conclusion:**

Our data show a curvilinear relationship between video game playing and mental health outcomes, with "moderate" gamers faring best and "excessive" gamers showing mild increases in problematic behaviors. Interestingly, "non-gamers" clearly show the worst outcomes. Therefore, both children and parents of non-game players should be updated about the positive impact of moderate video gaming. Educational interventions should also be designed to educate adolescents and their parents of the possible harmful impact of excessive video game playing on their health and psychosocial functioning.

## Background

Playing video games is now a major leisurely pursuit among children in many parts of the world [1-12]. Over the past three decades, a number of studies have looked at the effects of video games on children and adolescents. These studies were conducted mostly in developed high-income countries. Several of these studies have shown that violent video game exposure increases aggressive thoughts, angry feelings, physiological arousal, aggressive behaviors, and physiological desensitization to violence in the real world [1-11]. However, several other studies have found no connection between exposure to video game violence and youth violence [12-18]. Conducting a meta-analytic review of studies that examines the impact of violent media on aggressive behavior, Ferguson and colleagues (2009) reported that the use of poor aggression measures in most studies have inflated the effect size. Once corrected for methodological bias, they found no support for this notion that media violence leads to aggressive behavior [14]. Therefore, the evidence for the harmful effects of video game violence is, in fact, inconsistent.

Regardless, evidence suggests that the prevalence of video games, especially violent video games, among adolescents from low- and middle-income countries is increasing dramatically and requires additional investigation to evaluate the connection between violent video games and aggressive behaviors. Lack or insufficient reinforcement of copyright protections and the selling of rated video games to children in these countries have intensified public concerns on the possible negative impact of violent video games on aggressive cognitions, attitudes, behaviors, academic performance, and the psychological well-being of children. This study describes patterns and correlates of excessive video game use in a random sample of middle-school boys and girls in the Islamic Republic of Iran. Specifically, we examine the relationship between video game playing and psychological well-being, aggressive behaviors, and adolescents' perceived threat of video-computer game playing. To the best of our knowledge, no study has ever explored the prevalence and correlates of video games among children in Iran. The impact of violent video games on the attitudes, behaviors, and mental health of the youth in Middle-Eastern countries may be intensified or indeed suppressed by several ongoing violent conflicts in the region. Similar to the rest of Middle-Eastern countries, Iranians have constantly been exposed to well-publicized, broadcasted, excessive violence in the region, including an eight-year Iran-Iraq war, the first Persian Gulf war (Iraq vs. Kuwait and US-collection), the U.S. occupation of Afghanistan and Iraq following the tragedy of 9-11, almost 50 years of on-going Palestinian-Israeli conflict, as well as almost daily suicide and/or car bombings etc. by individuals in this region. Iran is the most populated country in the Middle East with a population of over 70 million. More than two-thirds of its population is under the age of 30 [19,20].

## Methods

### Sample

This cross-sectional study was performed with a random sample of eight schools selected from all 26 middle schools in the city of Hamadan, which is located in central Iran, with a population of over 500,000 people [21]. Almost 1950 sixth to eighth graders were registered in the eight randomly selected middle schools. One classroom from each school which includes 477 of adolescents (almost one out of four students) was selected to participate in this study. Determining the sample size for this study, we specified the difference between the largest mean and the smallest mean for GHQ-28 as the effect size (delta) and we hypothesized that all means for GHQ-28 other than the two extreme ones (non-gamers and excessive gamers) are equal to the grand mean. We calculated the sample size for three different effect size delta values (0.25, 0.75, 1.25) corresponding to "small", "medium", and "large" effects, according to Cohen & Cohen, Statistical Power Analysis for the Behavioral Sciences. The initial calculation indicates that using a medium effect size, we need at least 45 subjects in each group (180 participants) in order to detect a reasonable departure from the null hypothesis (α = 0.05 and 1 - β = 85%). However, our preliminary investigation showed that only 10% of students were non-players, therefore, if we would had selected 180 participants only 18 (180 × 10%) of them would be the non-players. Therefore, we increased the sample size by 250% (n = 180 × 250% = 450) to have 45 non-players in our sample.

This survey was self-administered in the absence of the instructor and collected by research associates of this project who were unaffiliated with the schools during regular classroom hours. To increase the validity of the responses, efforts were made to guarantee complete anonymity. Students were given a brief introduction and were asked not to write their name or any other identifiable information anywhere on the survey. The survey was conducted from February to March 2008. This study was conducted with approval from Hamadan University's Institutional Review Board. Informed assent and consent were obtained from participants and their parents/guardians. The survey instrument was pilot tested with 20 students and modified accordingly. The questionnaire was administered in Farsi, the official language of Iranian schools. Regardless of ethnicity or background, all Iranian students speak and write Farsi.

#### Measures

##### Demographics and video-game playing

In addition to demographic characteristics, survey instruments included several items that were specifically designed to capture the amount of time students spend playing video games during weekdays and weekends. Commitments to video game use, the average and longest duration of play, and the type of video games were assessed.

##### Mental health status

The validated 28-item Farsi version of the General Health Questionnaire (GHQ-28) [22] was used to assess the mental health status of participants. The GHQ refers to subjective symptoms of psychological distress, somatic manifestations often associated with anxiety and depression, relationship difficulties, and social, family, and professional roles [23]. The GHQ-28 is composed of 4 subscales (score range, 1-7): somatization, anxiety, social dysfunction, and depression. Both subscales and summated total scores were used [23,24]. All items have a 4 point scoring system using Likert scoring (0-1-2-3). A higher score on the GHQ-28 represents poorer mental health status. In our sample the Cronbach's alpha coefficients of reliability of the subscales vary around 0.75 and the internal consistency of the total scale is 0.90. The participants were classified into higher and lower GHQ-28 groups based on a cutoff score of 8.

##### Aggression

The Orpinas' aggression scale was used to measure aggressive behaviors of students [25]. The scale consists of 11 items designed to measure self-reported aggressive behaviors among school-aged students that might result in psychological or physical injury to other students. The scale requests information regarding the frequency of the most common overt aggressive behaviors, including verbal aggression (teasing, name-calling, encouraging students to fight, threatening to hurt or hit) and physical aggression (pushing, slapping, kicking, hitting), as well as information about anger (getting angry easily, being angry most of the day) [25]. The internal consistency of the scale showed coefficient alpha of 0.89.

##### Perceived side effects of video-computer games

A 5-item rating scale was used to gauge student beliefs on the side effects of playing excessive video games. The following are examples of the 5-item rating scale: "I believe that excessively playing video games has negative affects on educational performance;" and "I believe that obesity could be a side effect of excessively playing video games." Each of these items was measured on an ordinal 5-point Likert-type scale (1 = certainly disagree, 5 = certainly agree). Higher scores on the scale indicated a greater perceived threat of video-computer games. The internal consistency reliability of this scale was examined using Cronbach's coefficient alpha (α = 0.72).

##### Video violence exposure

Based on previous content analyses of popular video games among Iranian children, video games that were reported to be played by participants were categorized into two major groups: violent and non-violent games. Additionally, one of the co-authors of this study personally examined the content of each game to verify whether games are correctly categorized.

### Statistical Analysis

Descriptive statistics were calculated to identify the distribution and frequency of all items which were subsequently used to construct the independent scales and indices for examination. In the bivariate analysis, chi-square tests were performed to measure the association between independent and outcome variables. A series of logistic regression and descriptive analyses was computed. Interrelationships between independent variables were examined to assess potential multicolinearity between independent variables. All statistical analyses were performed using the statistical software package SPSS (SPSS Inc., Chicago, Illinois, USA).

## Results

Of 477 adolescents who were selected to participate in this study, 444 students (93%) completed the survey. Less than 7% of students refused to participate in the study or did not complete the survey. Since the survey was anonymous, we were unable to record and compare the non-responders (n = 31) with participants. Table 1 shows demographic and gaming characteristics, aggressive behaviors, and the mental heath status of our samples. About 47% and 53% of the participants were 12-13 and 14-15 years old, respectively. The sample was 51% female and 49% male. More than 93% of students who participated in this study reported that they have played video games. The initiation ages for video-game playing were 6%, 11%, 45%, and 40% for age groups ≤5; 6-7; 8-10; and 11-13 years of age, respectively. While computer games were used by 71%, home consoles by 16%, and internet games by 13%, no arcade video game machines were reported by our participants (the arcade video game machines currently are not offered in Hamadan).

**Table 1:** Characteristics of sample, comparing boys and girls.

Characteristics	Boys N (%) Mean ± SD	Girls N (%) Mean ± SD	Total N (%) Mean ± SD
Age			
12-13 years old	105 (48)	105 (47)	210 (47)
14-15 years old	115 (52)	119 (53)	234 (53)
Fathers' age	44 ± 6.73	45 ± 7.44	45 ± 7.35
Mothers' age	38 ± 5.74	38 ± 5.76	38 ± 5.74
Parents' highest education (college degrees)	53 (25)	60 (27)	113 (26)
Length of time playing video games per setting*	1.9 ± 1.19	1.8 ± 0.95	1.9 ± 1.07
Weekly hours playing video games*	6.7 ± 3.45	6.5 ± 3.08	6.6 ± 3.26
Years of video game playing*	2.9 ± 1.25	3.1 ± 1.20	3.0 ± 1.21
Initiation age for video gaming*	**10.2 ± 2.44**	**9.4 ± 2.46**	**9.8 ± 2.45**
Own bedroom (yes)	77 (35)	69 (31)	146 (33)
Personal computers (yes)	**136 (62)**	**118 (53)**	**254 (57)**
Severe arguments with parents (yes)	132 (61)	139 (62)	271 (62)
Self-reported aggressive behaviors	8.8 ± 8.28	9.1 ± 9.36	8.9 ± 8.83
Perceived side-effects of games	14.3 ± 4.01	14.0 ± 4.56	14.2 ± 4.29
General mental health status (GHQ-28)	**6.8 ± 6.20**	**4.6 ± 4.97**	**5.7 ± 5.72**
≤8	**143 (65)**	**182 (81)**	**325 (73)**
> 8	**77 (35)**	**42 (19)**	**119 (27)**
Somatic	**1.5 ± 1.68**	**1.0 ± 1.53**	**1.2 ± 1.62**
Anxiety/Insomnia	**1.8 ± 1.93**	**1.2 ± 1.56**	**1.5 ± 1.77**
Social dysfunction	**1.7 ± 1.70**	**1.3 ± 1.48**	**1.5 ± 1.60**
Severe depression	**1.9 ± 2.28**	**1.0 ± 1.53**	**1.4 ± 1.98**

*24 non-players excluded

More than 57% of children reported that they had a computer or game console in their bedroom. Both girls and boys spent an average of 6.3, and 6.2 hours per week playing video gaming, respectively. Moreover, 47% of participants reported that they had played one or more intensely violent games including: *Dead or Alive, Def Jam, Doom, Driver, Mortal Kombat, Grand Theft Auto, Resident Evil*, and *Prince of Persia*.

The bivariate correlates of video gaming are reported in Table 2. Participants' mental health status, measured by GHQ-28, showed a significant relationship with initiation age and years of video game playing, indicating that those who initiated gaming at younger ages and have been playing for longer years were more likely to score poorer in mental health measures. No relationship between severe arguments with parents and gaming was detected. Quite interesting were those participants who reported longer years and hours playing games - they also reported a higher number of the negative side effects of video gaming.

**Table 2:** Bivariate correlates of video gaming (n = 444).

Variables	X1	X2	X3	X4	X5	X6
X1 - Initiation age	1.00					
X2 - Length of time of playing video games	-.20**	1.00				
X3 - Years of video game playing	-.45**	27**	1.00			
X4 - Self reported aggression	-.07	.12*	.02	1.00		
X5 - Mental health status	.14**	.05	-.10**	.36**	1.00	
X6 - Perceived sides effect of video gaming	.08	-.17**	-.17**	-.03	.17**	1.00
X7 - Severe arguments with parents	-.02	.01	.01	.17**	.29**	.06

*p < .05; **p < .01; ***p < .001

Participants' aggressive behaviors were associated with length of gaming; those who admitted playing longer, scored higher on the Orpinas' aggression scale (r = .15; p < .05). Further analysis shows that none of the 11 items of this scale were associated with length of time playing video games. However, boys who reported playing video games excessively - more than 7 hours per week - scored higher on: 1) fighting back; 2) name-calling; 3) teasing; and 4) threatening to hurt or hit items. None of the aggressive behaviors scale's items were associated with excessive playing among girls.

Table 3 compares the mean score for the General Mental Health Status scale (GHQ-28) and the sub-scales of this index for four groups (non-players to excessive players). Non-gamers and "excessive gamers" overall reported suffering poorer mental health compared to low or moderate players. Additionally, non-gamers and "excessive gamers" reported higher scores on three out of four sub-scales, indicating that they were more likely than low or moderate players to suffer from anxiety/insomnia, social dysfunction, and somatic symptoms.

**Table 3:** Mean score of total and sub-scale scores on the 28-item General Health Questionnaire for video gamers and non-gamers (N = 444).

Scale/Subscale	Non-gamers	1-6 Hours/Week	7-10 Hours/Week	> 10 Hours/Week	**Sig**.
	(n = 24)	(n = 232)	(n = 120)	(n = 68)	
	Mean ± SD	Mean ± SD	Mean ± SD	Mean ± SD	
Somatic symptoms	1.96 ± 1.8	1.19 ± 1.6	1.04 ± 1.5	1.53 ± 1.7	< .05
Anxiety and insomnia	2.50 ± 2.4	1.41 ± 1.7	1.34 ± 1.7	1.82 ± 1.8	< .01
Social dysfunction	2.38 ± 2.0	1.42 ± 1.6	1.43 ± 1.3	1.53 ± 1.7	< .05
Severe depression	2.12 ±2 .5	1.40 ± 2.0	1.12 ± 1.6	1.73 ± 2.3	0.06
General mental health status	8.96 ± 7.7	5.43 ± 5.6	4.97 ± 5.0	6.67 ± 6.0	< .01

The multiple binary logistic regression technique was employed to examine independent correlates of excessive video game playing. Independent variables included in the equation are reported in Table 4. The estimated Nagelkerke R^2 ^indicates that this set of variables/subscales explain over 14% of the variance in the dependent variable. Age, personal computer ownership, and perceived side effects of playing video games each showed independent impact on the outcome variables. This finding indicates that, controlling for all other variables, older students [OR = 1.34 (95% CI = 1.04 - 1.74), p < .05], students who perceived less serious side effects for playing video games [OR = 3.5 (95% CI = 0.66 - 0.98), p < .01], and those who have personal computers [OR = 3.4 (95% CI = 2.13 - 5.41), p < .05] were more likely to report that they had played video games excessively. No significant independent relationship between parental demographic characteristics and excessive video gaming was detected.

**Table 4:** Multiple binary logistic regression between demographic characteristics, personal bedroom and computer ownership, and perceived side effect of gaming and excessive video game playing (n = 444).

Independent Variables	Odds Ratio	95% Confidence Intervals
**Age**	1.34*	**1.04 - 1.74**
Gender	0.89	0.58 - 1.37
Parents' education	1.05	0.83 - 1.32
Mother's working status	1.02	0.57 - 1.85
Personal bedroom	0.88	0.54 - 1.46
**Personal computer**	3.40***	**2.13 - 5.41**
**Perceived side-effects of video game playing**	0.81*	**0.66 - 0.98**

• *p < .05; **p < .01; ***p < .001

• Nagelkerke R-Squared = 0.14

• -2 Log Likelihood = 480.08

## Discussion

This cross-sectional study was performed to describe patterns and correlates of video game use in a random sample of middle-school students (aged 12 - 15) in the Islamic Republic of Iran. Similar to American teens [26], our data show that nearly every teen (93%) plays video games, regardless of gender, age, or socioeconomic status. Overall, our participants spent an average of 6.3 hours per week playing video gaming.

Additionally, more than 15% of participants spend more than 10 hours per week playing video games. The stereotype that only boys play video games is far from true in Iran, as girls constitute a larger percentage of total video gamers: 92% of boys play video games, as do 96% of girls. While almost all girls and boys play video games, boys typically play games with greater duration than girls per setting; 11% and 6% of boys and girls spend more than 4 hours per setting, respectively. One out of two adolescents surveyed in this study reported that they had played one or more intensely violent games.

One aspect of the results that warrants further discussion is the impact of the non-gaming and excessive video game playing on mental health status. Importantly, non-gamers and excessive gamers overall reported suffering poorer mental health compared to moderate players. As indicated in Table 4, we have detected a curvilinear relationship between video game playing and mental health outcomes, with "moderate" gamers faring best. While "excessive" gamers showed mild increases in problematic behaviors (somatic symptoms; anxiety and insomnia; social dysfunction, and general mental health status), non-gamers showed the worst outcomes. Other researchers have documented the same effect. Kutner and Olson (2008), surveyed 1,254 children in grades 7 and 8, and 500 of their parents. They found that boys who did not play any video games during a typical week had a higher risk for problems [27]. They also document many creative, social, and emotional benefits from video game play that were used by many children to relieve stress.

However, their surveys also documented statistically significant relationships between violent game play and some common childhood problems among boys but not girls. Boys who extensively played any Mature-rated game had twice the risk of certain aggressive behaviors and school problems compared to boys who played games with lower age ratings[27]. Indeed, similar to our study, several other studies suggest that moderate video game use may be a positive experience, while excessive use may cause problems [28]. While research suggests that that excessive use of video games may have a detrimental effect on students' GPA, moderate use of the video games and Internet has been linked with a more positive academic orientation when it is compared with nonuse or high levels users [29]. Therefore, if parents set some limits on their children's daily video game use, then the worst of the documented problems associated with video games will be avoided [28].

Another component of the results that requires further attention has to do with the detected impact of the perceived side-effects of video game playing on excessive game playing. The multiple binary logistic regression shows that controlling for all other variables, those who perceived less serious side effects for playing video games were more likely to report that they had played video games excessively. This important finding may have extensive policy implication. Educational motivational interventions should be designed to educate adolescents of the possible harmful impact of excessive video game playing on their health and psychosocial functioning.

Our findings suggest that playing video games may have different social implications for girls than for boys. Our data shows that boys, but not girls, who admitted playing video games excessively showed more aggressive behaviors. However, recent data show that moderate gaming among young men may provide a healthy source of socialization, relaxation, and coping [30]. In addition, recent studies conducted among Singaporean, Japanese, and Americans provide robust evidence that adolescents and young adults who played more pro-social video games behaved more pro-socially [31]. Therefore, strategies are needed to make pro-social video games more attractive and accessible to adolescents, particularly male adolescents. These results are not without their limitations that limit its generalizability. First, these data are cross-sectional and limit our ability to make causal inferences among the mental health status, aggressive behaviors, and video game playing. Second, we were unable to record and compare the non-responders of this study with participants and unable to document how this sample is a representative of Iranian adolescents. Third, it is important to notice that even though statistically a significant relationship between excessive gaming and mental health was detected, however, these bivariate correlations effect size fall below Ferguson's (2009) [32] recommendations for "practical significance" (r ≥ .20) and should not be over interpreted as strong evidence of harm.

## Conclusion

It is important to inform parents that while moderate video game use may be a positive experience, excessive use may cause problems. However, both children and parents of non-players should be updated about the positive impact of moderate video gaming. Similarly, comprehensive actions are needed in order to diminish excessive time spent playing video games, particularly mature-rated games, among adolescents in general and boys in particular. Finally, strategies are needed to encourage making pro-social video games more attractive and accessible to children, particularly male adolescents who play video games excessively.

## Competing interests

The authors declare that they have no competing interests.

## Authors' contributions

All authors read and approved the final manuscript. H.A. conceived of the study and participated in the design, data collection and analysis as well as MS preparation. M.B. participated in the data analysis and MS preparation. A.F. participated in the design and data collection. B.M. participated in the design data collection

## Pre-publication history

The pre-publication history for this paper can be accessed here:

http://www.biomedcentral.com/1471-2458/10/286/prepub
